# Green Fluorescent Protein (GFP)-Based Overexpression Screening and Characterization of AgrC, a Receptor Protein of Quorum Sensing in *Staphylococcus aureus*

**DOI:** 10.3390/ijms140918470

**Published:** 2013-09-06

**Authors:** Lina Wang, Chunshan Quan, Baoquan Liu, Yongbin Xu, Pengchao Zhao, Wen Xiong, Shengdi Fan

**Affiliations:** 1 Dalian Institute of Chemical Physics, Chinese Academy of Sciences, 457 Zhong-shan Road, Dalian 116023, China; E-Mails: wanglina.gucas@hotmail.com (L.W.); pengchao_zpc@163.com (P.Z.); 2 Department of Life Science, Dalian Nationalities University, Economical and Technological Development Zone, Dalian 116600, China; E-Mails: lbq@dlnu.edu.cn (B.L.); yongbinxu@dlnu.edu.cn (Y.X.); xiongwen-jan@hotmail.com (W.X.); fsd@dlnu.edu.cn (S.F.); 3 The State Ethnic Affairs Commission-Ministry of Education, Economical and Technological Development Zone, Dalian 116600, China

**Keywords:** membrane protein, detergent screening, GFP, IMAC, SEC, CD spectroscopy

## Abstract

*Staphylococcus aureus* AgrC is an important component of the *agr* quorum-sensing system. AgrC is a membrane-embedded histidine kinase that is thought to act as a sensor for the recognition of environmental signals and the transduction of signals into the cytoplasm. However, the difficulty of expressing and purifying functional membrane proteins has drastically hindered in-depth understanding of the molecular structures and physiological functions of these proteins. Here, we describe the high-yield expression and purification of AgrC, and analyze its kinase activity. A *C*-terminal green fluorescent protein (GFP) fusion to AgrC served as a reporter for monitoring protein expression levels in real time. Protein expression levels were analyzed by the microscopic assessment of the whole-cell fluorescence. The expressed AgrC-GFP protein with a *C*-terminal His-tagged was purified using immobilized metal affinity chromatography (IMAC) and size exclusion chromatography (SEC) at yields of ≥10 mg/L, following optimization. We also assessed the effects of different detergents on membrane solubilization and AgrC kinase activity, and polyoxyethylene-(23)-lauryl-ether (Brij-35) was identified as the most suitable detergent. Furthermore, the secondary structural stability of purified AgrC was analyzed using circular dichroism (CD) spectroscopy. This study may serve as a general guide for improving the yields of other membrane protein preparations and selecting the appropriate detergent to stabilize membrane proteins for biophysical and biochemical analyses.

## 1. Introduction

*Staphylococcus aureus* is an invasive pathogen that causes various human illnesses, ranging from skin infections and food poisoning to toxic shock syndrome and endocarditis [1]. The pathogenicity of *S. aureus* is highly complex due to the secretion of virulence factors secreted by the bacteria. The secretion of virulence toxins is controlled by the accessory gene regulator (*agr*) quorum sensing system that is composed of an autoinducing peptide (AIP) and a two-component signal transduction system (TCST) [2,3]. The TCST is commonly found in prokaryotes, fungi and plants, and it serves to sense and respond to external environment stimuli that are related to survival [4–7].

In *S. aureus*, the *agr* quorum sensing system contains genes of a typical TCST system, which controls many physiological processes. The TCST system in *S. aureus* is comprised of the membrane-bound histidine kinase (HK) sensor AgrC, which responds to the AIP signaling molecule, and the cytoplasmic response regulator (RR) AgrA, which gives rise to a cellular response [8]. *S. aureus* has four diverse *agr* alleles. In general, only the cognate ligand–receptor interaction induces activation, whereas most heterologous interactions inhibit the receptor activation. Recent reports have shown that the first and second extracellular loops of AgrC facilitate AgrC activation [9–12]. Additionally, Geisinger *et al.* identified amino acid I171 in the third extracellular loop of AgrC to be critical for ligand-receptor recognition [13]. However, the mechanism in *S. aureus* for sensing and responding to AIP and the specific sites of AgrC oligomer formation remain largely unknown. To resolve these issues at the molecular level and gain insight into the structure of the AgrC, it is important to express and purify a sufficient amount of functional full-length AgrC. AgrC is a 430-residue intra-membrane sensor kinase, the topology of which includes an *N*-terminal sensor domain (residues 1–204 for AgrC), with six to seven transmembrane helices and a cytoplasmic domain (residues 205–430).

The difficulty of expressing and purifying functional membrane proteins has drastically hindered structural and functional studies of these proteins. A *C*-terminal green fluorescent protein (GFP) fusion to a target protein is commonly used as a reporter for monitoring expression and purification [14–17]. Therefore, we constructed a recombinant plasmid pET-28-AgrC-GFP to express AgrC in *Escherichia coli*. In addition, we optimized the quantity of correctly folded protein by adjusting several parameters, including the host cells, growth culture medium, inducer concentration, and induction temperature.

Here, we describe, for the first time, a GFP fluorescence-based approach to assess the expression, purification, and detergent solubility of full-length AgrC from the *agr-*I allele. In addition, we also describe the effect of different detergents on AgrC kinase activity. Stabilization of AgrC secondary structure after purification was assessed by CD spectroscopy. Although our primary model system is based on the production of the *S. aureus* AgrC, a similar approach can be used to overexpress other recombinant proteins. This study provides a basis for designing rational strategies to improve the yields of membrane protein preparations, and these findings also provide the first biochemical data on AgrC.

## 2. Results and Discussion

### 2.1. Expression Screening and Optimization

Overexpression of membrane proteins remains a major challenge for biochemical and structural research. While many alternative organisms and expression systems are also used to produce recombinant proteins, *E. coli* is the most common. GFP, as previously reported, is an excellent indicator for the simultaneous detection of proper protein folding and aggregation [18,19]. We took advantage of this GFP-based screening system to evaluate and monitor the expression level of membrane protein AgrC in *E. coli* host cells.

**Figure 1:**
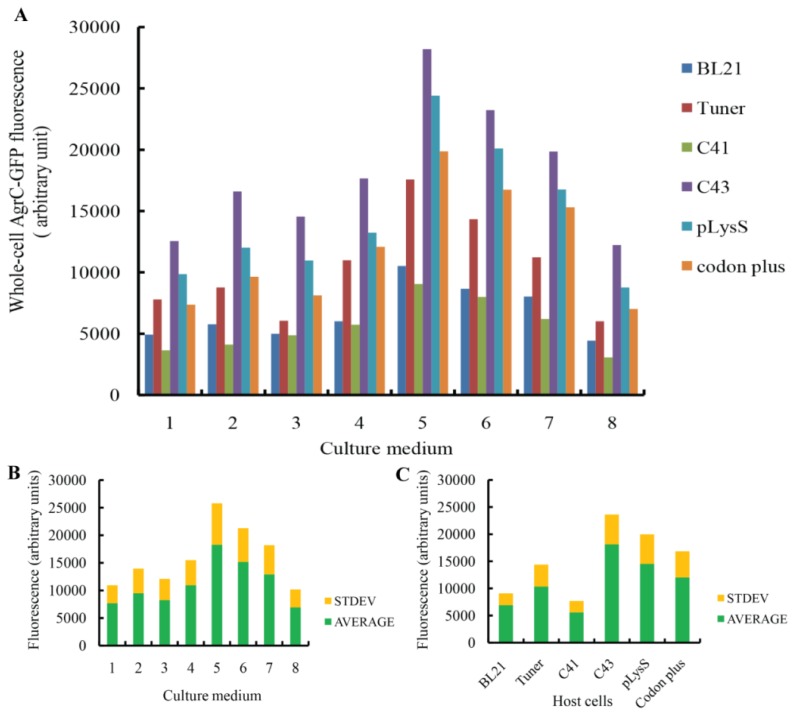
Screening of AgrC-green fluorescent protein (GFP) expression in different host cells and culture media. (**A**) Whole-cell fluorescence of different host strains. Screening of optimal expression conditions using a combination of eight different types of media (LBE, LBE-LP, LBE-MP, 2× TYE, TBE, 32Y, 4× TY, and TB) and six *E. coli* strains (BL21, Tuner, C41, C43, pLysS, and codon plus) at 20 °C. Protein expression was induced with 0.1 mM IPTG. Fluorescence counts were recorded from 200 μL cell cultures. This experiment was repeated at least three times, and the results were reproducible; 1 = LBE, 2 = LBE-LP, 3 = LBE-MP, 4 = 2× TYE, 5 = TBE, 6 = 32× Y, 7 = TB, 8 = 4× TY; (**B**) Statistical significance of the fluorescence counts for each of the six cell strains; (**C**) Statistical significance of the fluorescence counts for each of the eight types of media. The green bars indicate average values and the yellow regions correspond to standard deviations.

To improve the yield of our protein overexpression system, an initial screening was performed in triplicate with six different *E. coli* strains (BL21(DE3), Tuner(DE3), C41(DE3), C43 (DE3), BL21 (DE3)-pLysS, and BL21-CodonPlus (DE3)-RIL) and eight different types of media (LBE, LBE-LP, LBE-MP, 2× TYE, TBE, 32Y, TB, and 4× TY). Expression efficiency was compared using a fluorescence assay that measures GFP fluorescence intensity in 200 μL of cells from each of the 48 combinations of protein expression conditions, generating a wide distribution of fluorescence signals with values of 3000–30,000 fluorescence arbitrary units (F.A.U.). Induced host cells containing an empty expression plasmid that were grown to a similar density yielded a background value of approximately 2500 F.A.U. We successfully induced protein expression using all of the combinations of host cells and media. The combination of the C43 strain with the TBE medium yielded the optimal expression of AgrC-GFP among all combinations (Figure 1A). As shown in Figure 1B,C, the average fluorescence intensity of C43 was the highest among all strains, while that of TBE was also the highest among the various culture media. Thus, this combination resulted in the maximum production of AgrC-GFP in the native state. This finding also agrees with previous studies showing that C43 is the most tolerant strain to the toxicity of expressed membrane proteins [18,20] and that TBE was the most nutrient-rich medium [21,22]. Therefore, we used *E. coli* C43 and TBE medium for subsequent AgrC expression experiments.

**Figure 2:**
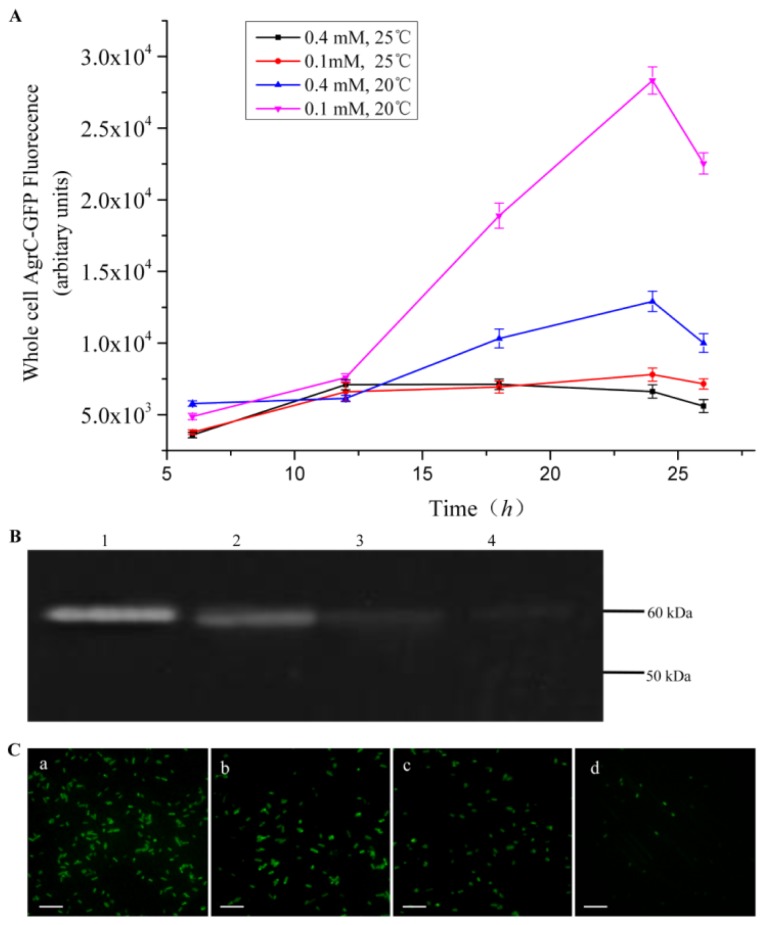
Screening of AgrC-GFP expression under different temperatures and inducer concentrations. (**A**) Monitoring of whole-cell fluorescence intensity from cells overexpressing AgrC-GFP every 6 h after IPTG induction. Black, red, green, and blue lines represent four induction conditions, including 0.4 mM IPTG at 25 °C; 0.1 mM IPTG at 25 °C; 0.4 mM IPTG at 20 °C; and 0.1 mM IPTG at 20 °C. The experiments were performed in triplicate; (**B**) In-gel fluorescence detection for AgrC-GFP expression. The lanes were loaded as follows: (1) 0.1 mM IPTG, 20°C; (2) 0.4 mM IPTG, 20 °C; (3) 0.1 mM IPTG, 25 °C; and (4) 0.4 mM IPTG, 25 °C; (**C**) Fluorescence microscopy images of cells expressing AgrC-GFP. *E. coli* C43 cells were induced under different conditions as indicated above the panel; (a) 0.1 mM IPTG, 20°C; (b) 0.4 mM IPTG, 20 °C; (c) 0.1 mM IPTG, 25 °C; and (d) 0.4 mM IPTG, 25 °C. The scale bar is 10 μm. For each sample, an approximately equal number of cells were captured.

Protein expression was induced at 20 °C or 25 °C with 0.1 mM or 0.4 mM isopropyl-β-d-thiogalactoside (IPTG). The optimal induction condition for AgrC was found to be 20 °C and 0.1 mM IPTG (Figure 2A). AgrC expression at a low temperature and low inducer concentration may help prevent premature aggregation leading to inclusion body formation and may ensure proper membrane insertion. More elaborate schemes in which cell growth was performed at different temperatures (16 °C and 18 °C) produced the same result as at 20 °C but required a longer induction time (data not shown). Previously, the applicability of GFP as a reporter protein for monitoring the proper folding of soluble proteins has been assessed based on whole-cell GFP fluorescence intensity [17]. We examined whether in-gel GFP fluorescence of the cellular protein extract would be consistent with whole cells fluorescence of *in vivo*. An advantage of in-gel fluorescence measurement is the ability to verify the integrity of GFP-fusion proteins. After cell lysis, the total protein extracts of different cultures that were induced under various conditions were subjected to SDS-PAGE. The gel was rinsed with double distilled water, and fluorescence was measured directly using a fluorescence imaging system (Figure 2B). In-gel fluorescence intensity was found to be higher at lower temperatures and inducer concentrations, which agreed with the results of whole-cell fluorescence measurement. For whole-cell measurement, *E. coli* C43 cells expressing AgrC-GFP were imaged using a fluorescence microscope (Figure 2C). As expected, the host cells displayed green fluorescence at their periphery. The fluorescence data demonstrated that the most suitable induction condition was 0.1 mM IPTG and 20 °C.

### 2.2. Detergent Evaluation Based on GFP Fluorescence and Kinase Activity

Membrane proteins reside in a very complex environment, making them difficult to study *in situ*. Therefore, a detergent that not only disintegrates native membranes but also closely mimics biological conditions is necessary to maintain a purified protein in its native state for as long as possible. Although several detergents are commonly used in molecular biology research, a universal detergent for purifying all membrane proteins would be ideal. In this study, we screened a number of detergents to identify the most suitable choice for purifying AgrC.

**Figure 3:**
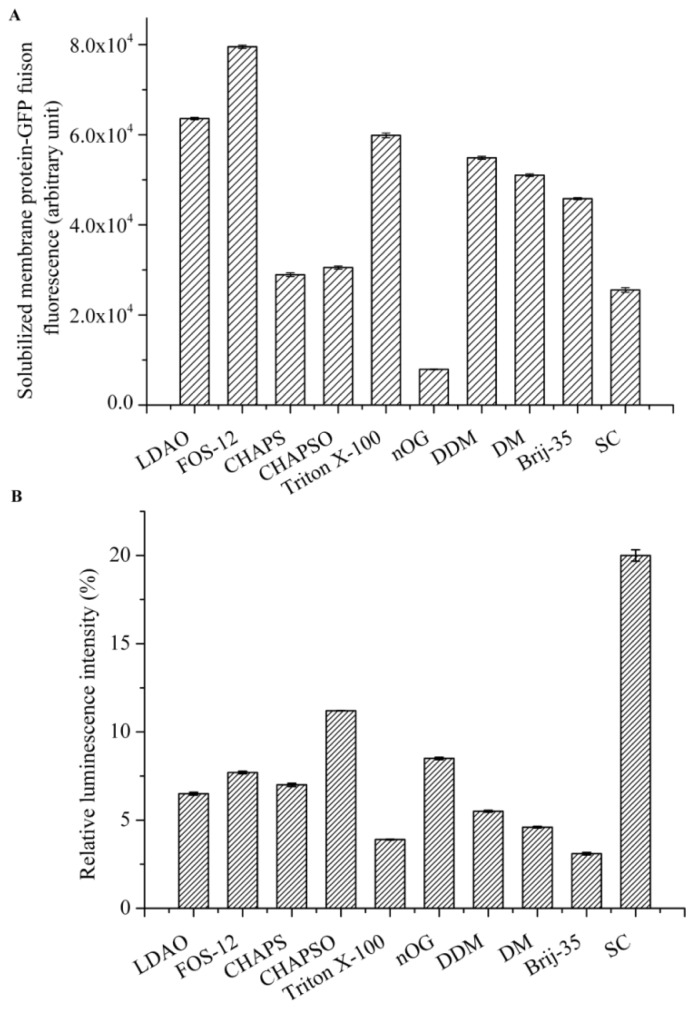
Detergents screening based on degree of cell membranes dissolution and level of kinase activity. (**A**) Comparison of different detergents for their ability to efficiently solubilize cell membranes. Different detergents (10× critical micelle concentration (CMC)) were separately added to cell membrane suspensions. After ~2 h of solubilization on ice, the suspensions were centrifuged at 200,000× *g* for 50 min at 4 °C, and then GFP fluorescence intensity of the 200 μL supernatant aliquots were measured on a spectrofluorometer; (**B**) The effect of different detergents on AgrC kinase activity. As a negative control, a kinase reaction was performed in the absence of AgrC. Luminescence was recorded on a Synergy2 Multi-Mode Microplate Reader. Relative luminescent intensity was expressed as a % control reaction. Values represent the mean ± S.D. of three replicates of three independent experiments.

Based on solubilization of cell membranes, we found that Fos-choline-12 (Fos-12) was the most effective detergent for solubilization (Figure 3A). *N*,*N*-dimethyldode-cylamine *N*-oxide (LDAO) was the second-most effective, followed by Triton X-100, *n*-dodecyl-β-d-maltopyranoside (DDM), n-decyl-β-d-maltopyranoside (DM), polyoxyethylene-(23)-lauryl-ether (Brij-35), 3-[(3-cholamidopropyl)dimethylammonio]-2-hydroxy-1-propanesulfonate (CHAPSO), 3-[(3cholamidopropyl)-dimethylammonio]-1-propane sulfonate (CHAPS), and sodium cholate (SC). n-octyl-β-d-glucopyranoside (nOG) was the least effective for membrane solubilization. However, we identified the most appropriate detergent not only for its ability to solubilize membranes but also for its ability to retain the kinase activity. We measured kinase activity by quantifying the amount of ATP that remained in solution after an *in vitro* phosphorylation reaction (Figure 3B), which was inversely correlated with the kinase activity of AgrC. Noticeably, AgrC in the presence of detergent Brij-35 exhibited the highest activity. Triton X-100 ranked second, followed by DM, DDM, LDAO, CHAPS, FOS, nOG, and CHAPSO. AgrC kinase activity was the lowest in SC, which may be due to protein destabilization in this strongly ionic detergent. This finding is consistent with the idea that ionic detergents are more destructive towards membrane proteins [23]. FOS-12 and LDAO are zwitterionic detergents. Compared with nonionic detergents, they were better at solubilizing membranes but worse at retaining kinase activity. One reason may be protein denaturation under harshly ionic conditions. DDM was more effective than DM for membrane solubilization, and this may be because of the methyl group distinguishes their tails, even though they share the same maltoside heads. We also observed that the nonionic detergent nOG and the moderately strong detergents, including CHAPSO and CHAPS, resulted in lower extraction efficiency and kinase activity.

In summary, we found that there is no conventional rule that can be applied to the selection of the best detergent for stabilizing AgrC *in vitro*. Therefore, the degree to which a detergent can best simulate the membrane bilayer depends on both the particular detergent and the particular protein [24]. Our data may serve as a basic guide for the screening of major classes of detergents to identify the best choice for extracting particular membrane proteins from lipid environments. If the goal is to maintain membrane protein activity, a mild detergent may be a better choice, such as Brij-35 and Triton X-100. Additionally, it may be necessary to select an appropriate combination of detergents and find new or generic detergents for use in biochemical studies of integral membrane proteins.

### 2.3. Stimulation of Autophosphorylztion in Purified AgrC by the Signalling Molecule AIP

**Figure 4:**
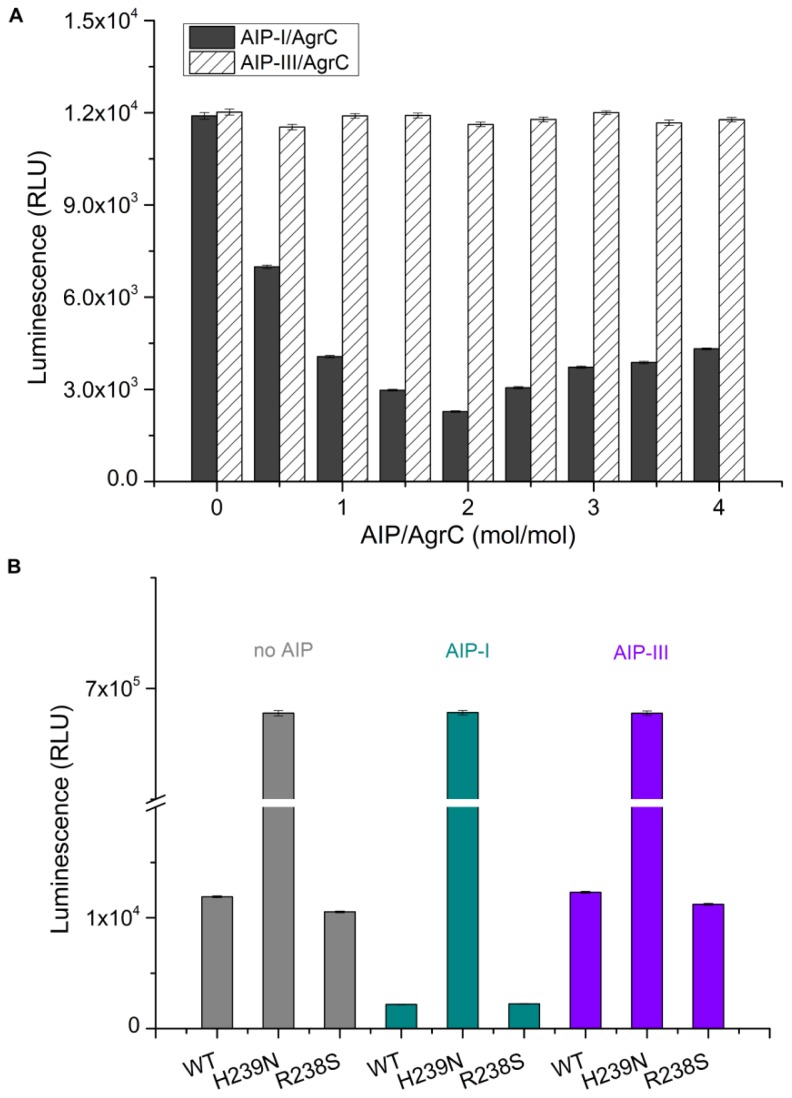
Effect of the AIP-I and AIP-III on the autophosphorylation of AgrC and AgrC mutants. Each autophosphorylation reaction contained 100 μg of purified AgrC or AgrC mutant that had been preincubated in 50 μL of reaction buffer in the presence or absence of AIP-I or AIP-III. After 20 min of kinase reaction, an equal volume of Kinase-Glo kit reagent was added. Luminescence was recorded on a Synergy2 Multi-Mode Microplate Reader. (**A**) AgrC autophosphorylation levels in response to increasing concentrations of AIP-I or AIP-III; (**B**) AgrC mutant autophosphorylation levels with or without AIP-I or AIP-III. Values represent the mean ± S.D. of three replicates from three independent experiments.

The virulence level in *S. aureus* is regulated via *agr*-dependent quorum sensing, in which the signaling molecule AIP-I activates AgrC from the *agr-*I allele, whereas AIP-III is inhibitory [10]. For this study, AIP-I and AIP-III were synthesized chemically in its active form. To demonstrate that synthetic AIP-I can specifically induce AgrC activation, purified AgrC was pre-incubated with different concentrations of AIP-I and AIP-III for 30 min prior to the autophosphorylation assay. In the kinase assay, the amount of ATP remaining in solution following a kinase reaction was measured using Kinase-Glo and luminescence is inversely related to kinase activity. As shown in Figure 4A, AIP-I increased AgrC autophosphorylation by approximately five-fold when the molar ratio of AIP-I to AgrC was 2:1, and higher AIP-I concentrations elicited little increase in the level of phosphorylated AgrC. Meanwhile, AIP-III had little effect on the level of AgrC autophosphorylation, regardless of its concentration (Figure 4A). We next examined the effect of AIP-I and AIP-III on a non-functional AgrC mutant (H239N) and a constitutively active AgrC mutant (R238S). As expected, the nonfunctional AgrC mutant (H239N) lost the kinase activity, regardless of the presence of AIP-I or AIP-III (Figure 4B). In contrast, the active AgrC mutant (R238S) retained the constitutive kinase activity. Using a two-fold molar excess of AIP-I, the AgrC (R238S) mutant showed a five- to six-fold increase in activity; in addition, it was unaffected by AIP-III. These results confirmed that AIP-I exerts a significant and specific effect on AgrC activity. These data also provided the first evidence for a direct and specific interaction between AIP-I and AgrC.

### 2.4. Purification of AgrC-GFP Based on IMAC and SEC

**Figure 5:**
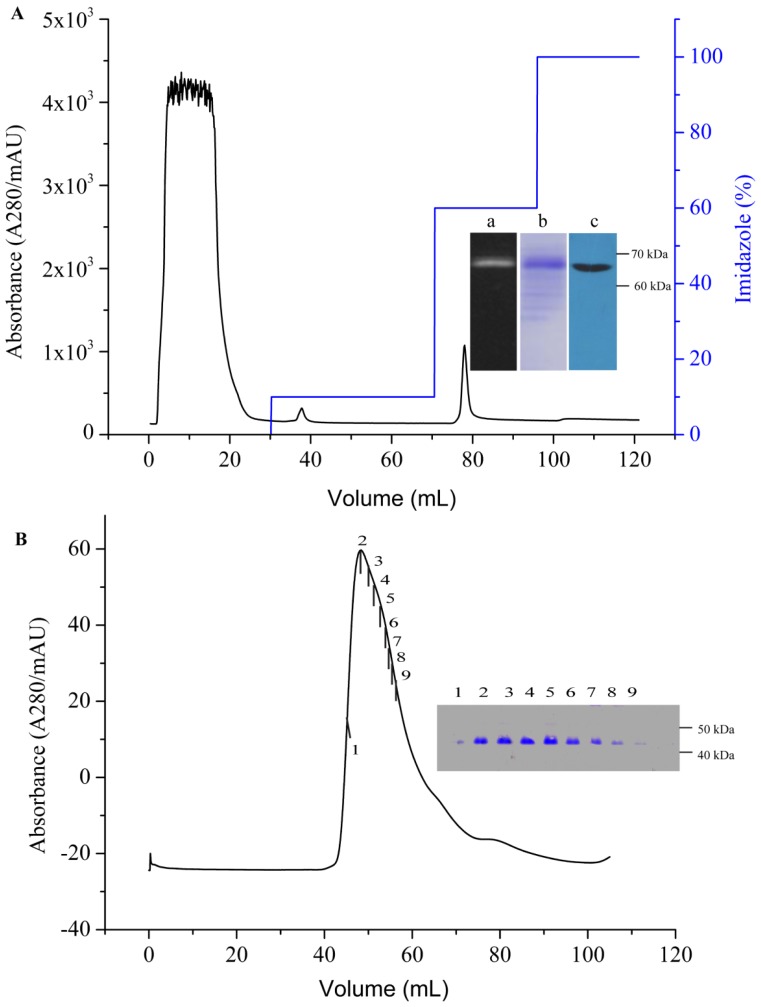
Protein purification. (**A**) Preliminary purification of AgrC-GFP by immobilized metal affinity chromatography (IMAC). The fraction that was eluted from the IMAC column (packed with 5 mL of HisTrap HP) with 60% elution buffer using an ÄKTA express system was analyzed by SDS-PAGE. (**a**) In-gel GFP fluorescence image; (**b**) staining of the same part of the gel with Coomassie brilliant blue; and (**c**) immunoblotting of the same part of the gel with anti-His-HRP; (**B**) Superdex200 SEC profiles. Following TEV digestion and batch IMAC purification, the sample was subjected to SEC and an SDS-PAGE analysis of the SEC peak is shown in the inset. The numbers 1–9 corresponding to the fractions on the chromatogram. The molecular weight was calculated to be ~47 kDa, which matched the theoretical molecular weight of native AgrC.

To confirm that the reported expression levels and detergent selections are scalable for downstream structure-function applications, the fusion protein AgrC-GFP was purified by immobilized metal ion affinity chromatography (IMAC) using the best detergent identified at the detergent screening stage (Figure 3A,B). By monitoring A_280_ absorption peaks, we effectively collected AgrC-GFP from a solution of Brij-35. As shown in Figure 5A, AgrC-GFP was eluted using 60% elution buffer containing 300 mM imidazole. Protein purity was determined by Coomassie-staining of gel after SDS-PAGE and western blot (Insets a, b, and c of Figure 5A). The molecular weight of AgrC-GFP was calculated to be ~74 kDa. Even though the observed bands for AgrC–GFP were not at the calculated molecular weight, this could be due to the structural conservation of the GFP fraction [18]. IMAC is a common protein purification method, with the advantages of strong, specific binding, and gentle elution conditions [25]. AgrC–GFP containing a tobacco etch virus (TEV) protease recognition site was digested with TEV protease at 16 °C for 5 h to remove the GFP-His6 tag. After TEV digestion, the cleaved sample was incubated with IMAC nickel resin and the flow-through containing AgrC was collected. Further separation and purification of AgrC was achieved by size-exclusion chromatography (SEC) (Figure 5B). The monodispersity and purity of the protein solution were analyzed using SDS-PAGE (Inset in Figure 5B). The purity of the eluted fraction was at least 95%. Finally, the concentration of purified AgrC was determined by BCA assay to be ≥10 mg/L. Generally speaking, the GFP tag should be removed from a fusion protein to allow further studies of protein stability and function, even though a GFP tag has many advantages during protein expression and purification. However, tag removal may be problematic due to improper folding of the fusion protein in a detergent micelles and the inhibitory effects of some detergents on the proteolytic enzyme that is used [26,27]. To avoid such problems, we used a GFP-free vector pET-28a-AgrC for medium or large scale expression and purification of AgrC. Our results showed that the GFP-free vector yielded comparable levels of AgrC expression and kinase activity as the GFP-fusion vector, if not higher, under the same optimized conditions that we described above (results not shown). It has also been reported previously that there is a good correlation between GFP-free protein expression and GFP-fused protein expression in high throughput screening [28].

### 2.5. Secondary Structure and Structural Stabilization of Purified AgrC

**Figure 6:**
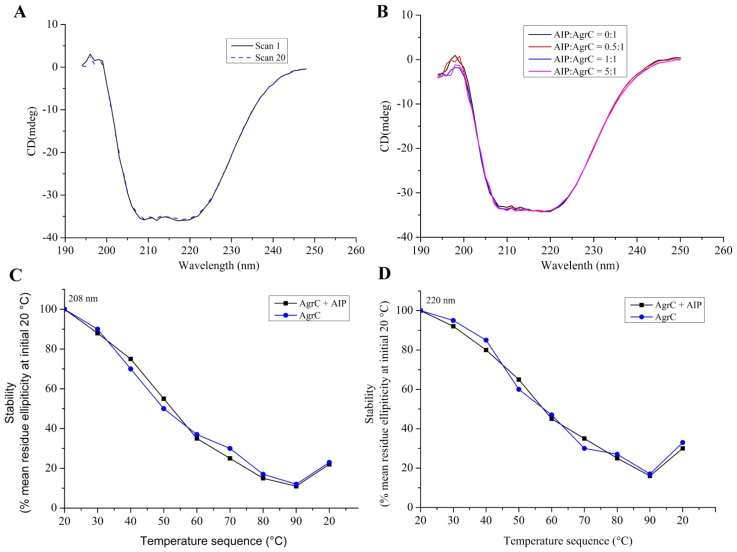
Determination of AgrC stability from CD measurements. (**A**) Far UV spectra of purified AgrC before and after exposure to far UV radiation following sample preparation in 10 mM 4-(2-hydroxyethyl)-1-Piperazineethanesulfonic acid (HEPES) (pH 7.4) containing 10× CMC Brij-35, 100 mM NaCl, and 10% glycerol at 20 °C. Spectrum 1, immediately after stabilization (solid line); spectrum 20, after 1 h exposure to light radiation (190–250 nm) during 19 consecutive scans (dashed line); (**B**) Spectra of stabilized AgrC in the presence of AIP-I. Data from each individual scan (unsmoothed) are shown; (**C** and **D**) Thermal stability of AgrC in the presence or absence of two-fold molar excess of AIP-I was expressed as % initial mean residue ellipticity obtained at 20 °C and (**C**) 208 nm or (**D**) 220 nm.

Although the kinase activity assays (Figure 4) demonstrated that AgrC was prepared as an active protein, retention of secondary structural integrity by our AgrC preparation method needed to be further investigated using CD in the far-UV range. The spectra that we obtained revealed a typical α-helical membrane protein (Figure 6A), thereby confirming the retention of AgrC structural integrity. The purification procedure that we adopted was, therefore, suitable for obtaining a correctly folded protein, with long-tern stability. Reproducibility and stability were further confirmed in comparisons of an initial spectrum of freshly stabilized AgrC with that obtained following a 1 h exposure to 190–250 nm radiation during 19 repeated scans on the CD instrument.

The effect of AIP-I on the secondary structure of AgrC was also investigated using CD in the far-UV (190–250 nm) region. Figure 6B shows that the addition of a 0.5- to 5-fold molar excess of AIP-I exerted little effect on the AgrC spectrum. Thermal studies revealed similar data in the presence or absence of the AIP-I ligand (Figure 6C,D). Comparisons of the peaks (208 nm and 220 nm) at different temperatures revealed that, when the temperature was higher than 30 °C, AgrC was less stable in the presence of Brij-35, just retaining 10%–20% of secondary structure at 90 °C (Figure 6C,D). Temperature-associated secondary structural damage seemed to be irreversible, because a return to 20 °C resulted in a negligible recovery of structural integrity. In conclusion, AIP-I had no detectable effect on the thermal stability of AgrC. Although micelles can at best partially mimic the native membrane environment of AgrC, they are largely insufficient to emulate the *in vivo* conditions of cell membranes. Therefore, to fully understand whether AIP-I can affect the secondary structure, full-length AgrC must be studied in a real membrane milieu. To accomplish this, AgrC should be incorporated into native-like membrane systems for further biophysical and biochemical analyses.

## 3. Experimental Section

### 3.1. Plasmid Construction

Before inserting AgrC into the expression vector, the location of the *C*-terminus was confirmed to be in the cytoplasm using the online programs TMHMM and TOPCONS, which are common methods for predicting membrane protein topology [29–31].

The *agr*C gene was amplified using primers containing *Nco*I (Forward: 5′-**GGGGG**CCATGGAATTATTAAATAGT-3′) and *Bam*HI (Reverse: 5′-**GGT**GGATCCTTTGTTTAGTATTAG-3′) restriction endonuclease recognition site with *S. aureus* chromosomal DNA used as the template. Restriction sites for *Nco*I and *Bam*HI are underlined in the forward and reverse primers, respectively. The thermal cycling procedure included one cycle at 95 °C for 5 min, 30 repeated cycles at 95 °C for 30 s, 50 °C for 30 s, and 72 °C for 1 min, and 1 cycle at 72 °C for 10 min. The resulting PCR product was cloned into the pMD19-T vector (Takara, Shiga, Japan), and then the AgrC fragment was subcloned into the pET-28a vector using the restriction sites for *Nco*I and *Bam*HI, whereas the *GFP* gene was amplified from the pEGFP plasmid (Clontech, Mountain View, CA, USA). The *GFP* gene fragment was then cloned into pET-28a-AgrC using the restriction endonucleases *Bam*HI and *Hin*dIII, generating the expression plasmid pET-28a-AgrC-GFP (Figure S1). The fusion protein carried a *C*-terminal 6× His tag. The AgrC mutants (R238S and H239N) were constructed by PCR using the QuickChange Site-Directed Mutagenesis Kit (Stratagene, La Jolla, CA, USA). DNA sequencing was used to verify the sequences of the constructed vectors.

### 3.2. Expression Screening of AgrC–GFP

To produce the AgrC–GFP fusion protein, the expression vector was introduced into the *E. coli* strains BL21(DE3), Tuner(DE3), C41(DE3), C43 (DE3), BL21 (DE3)-pLysS, and BL21-CodonPlus (DE3)-RIL. These host cells were grown in the nutrient-rich medium 32Y and several modified media, including LBE, LBE-LP, LBE-MP, 2× TYE, TBE, TB, and 4× TY (Table 1) [17,21–31]. Forty-eight different conditions were designed to screen for optimal combinations of medium and host strain. All combinations were cultured at 30 °C. For BL21 (DE3), Tuner (DE3), BL21 (DE3)-pLysS, and BL21-CodonPlus (DE3)-RIL cells, protein expression was induced with 0.1 mM IPTG at 20 °C after the cells reached an OD600 of ~0.5. For C41 (DE3) and C43 (DE3) cells, protein expression was induced at OD600 of 0.25–0.35 [16]. Induced host cells containing an empty expression plasmid were used as negative controls.

Comprehensive screening methods were developed to optimize protein expression levels. Specifically, we modified the inducer concentration and temperature. Duplicate cultures were grown at 20 °C or 25 °C, and protein expression was induced with 0.1 mM or 0.4 mM IPTG. We selected the combination of C43 and TBE for this screen because it produced the highest amount of AgrC among all the combinations of host cells and culture media. All cells were grown aerobically in 500-mL baffled shake flasks, each containing a 50-mL working volume with appropriate antibiotics. To monitor protein expression levels in real-time, we removed 200 μL of culture for whole-cell fluorescence measurements every 6 h.

### 3.3. Fluorescence Measurement

The fluorescence intensity of cultured cells was measured at regular intervals throughout the experiment using a fluorescence spectrophotometer (BioTeK, Winooski, VT, USA). Accumulation of the fusion protein AgrC-GFP was detected using an excitation wavelength of 485 nm and an emission wavelength of 510 nm. For fluorescence microscopy, cultures were diluted in ice-cold phosphate-buffered saline (PBS) to an OD_600_ of approximately 1.0 and then were mounted on a slide. Images were processed using DPcontroller ver. 2.2.1.227 software (Olympus, Tokyo, Japan).

### 3.4. Cell Disruption

After protein expression, cells were harvested by centrifugation at 8000× *g* for 10 min at 4 °C and washed twice with ice-cold PBS. Collected cells were resuspended in PBS (approximately 1.5 g of wet weight cells per 10 mL of PBS) containing MgCl_2_, imidazole, pefabloc SC, protease inhibitor cocktail, and DNase at final concentrations of 1 mM, 20 mM, 1 mg/mL, 1 tablet/50 mL, and 100 U/mL, respectively. Resuspended cells were rocked for 2 h on ice, followed by lysis through a High Pressure Homogenizer (JN-3000 PLUS) at a pressure of 800 bar at least three times at 4 °C. Lysed cells were subjected to centrifugated at 24,000× *g* for 20 min at 4 °C to remove unbroken cells and debris. The membranes were pelleted by ultracentrifugation at 300,000*× g* for 1 h and then solubilized by agitation in PBS buffer with the addition of detergent at 10× CMC and 10 mM imidazole for 1 h. Insoluble material was removed by ultracentrifugation at 200,000*× g* for 1 h.

### 3.5. SDS-PAGE and Western Blotting Analysis

Expressed proteins were subjected to SDS-PAGE, and then the SDS gel was rinsed with deionized H_2_O. Fluorescent bands were detected using a CCD camera system. Proteins were transferred from the gel onto a polyvinylidene difluoride (PVDF) membrane with a constant current of 90 mA for 1 h. The membrane was blocked with 1% non-fat milk powder in PBS buffer containing 0.05% (*v*/*v*) Tween-20. Mouse anti-His-tag antibody (SangonBiotech, Shanghai, China) was used as the primary antibody at a 1:2000 dilution in blocking solution. Rabbit-anti-Mouse horseradish peroxidase (HRP)-conjugated secondary antibody (SangonBiotech, Shanghai, China) was used at a dilution of 1:3000. Protein bands were detected on photographic films using an enhanced chemiluminescent substrate. Experiments were conducted with independent triplicate samples.

### 3.6. Detergent Selection

Detergent screening is vital for the effective solubilization of membrane proteins from the native membrane. We focused on detergents that are commonly used for the solubilization, purification, and crystallization of membrane proteins, including n-octyl-d-glucopyranoside (nOG), n-decyl-β-d-maltopyranoside (DM), n-dodecyl-β-d-maltopyranoside (DDM), Fos-Choline-12(Fos-12), C12E23 (Brij-35), 3-[(3-cholamidopropyl)-dimethylammonio]-1-propane sulfonate (CHAPS), 3-[(3-cholamidopropyl) dimethylammonio]-2-Hydroxy-1-Propanesulfonate (CHAPSO), *N*,*N*-dimethyldode-cylamine *N*-oxide (LDAO), sodium cholate (SC), and Triton X-100. All detergents were purchased from Sigma (St. Louis, MO, USA) at the highest purities available.

Cell membranes that were suspended in PBS were adjusted to a protein concentration of 10 mg/mL and the following detergents were evaluated: Fos-12, LDAO, CHAPS, CHAPSO, DDM, DM, nOG, Brij-35, Triton X-100, and SC typically at 10× CMC. Solubilization was carried out by gentle agitation for 2 h on ice. After centrifugation at 200,000× *g* for 50 min at 4 °C, the supernatant containing the solubilized AgrC-GFP was collected, and the best detergent for membrane solubilization was determined by measuring GFP fluorescence in 200 μL of the supernatant.

The intrinsic kinase activity of AgrC in various detergent micelles was measured by quantitating the amount of ATP remaining in solution following a kinase reaction using Kinase-Glo^®^ Luminescent Kinase Assay Kit (Promega, Fitchburg, WI, USA). The assay was performed in 96-well white plates in 50 μL kinase reaction volumes containing 100 μg AgrC and 5 μM ATP in assay buffer consisting of 10 mM HEPES (pH 7.4), 10 mM MgCl_2_, 50 mM KCl, and 10× CMC detergent. Negative controls were reaction mixtures containing no AgrC. The kinase reaction mixture was incubated at 37 °C for 20 min. Following incubation, 50 μL of ATP detection reagent was added to the assay plates. The plates were incubated at 37 °C for another 10 min, and relative light unit (RLU) signal was collected using the Synergy2 Multi-Mode Microplate Reader (BioTek, Winooski, VT, USA). The luminescent signal was positively correlated with the amount of ATP present and is inversely correlated with the amount of kinase activity.

### 3.7. Purification of AgrC–GFP

An immobilized metal affinity chromatography (IMAC) column (HisTrap HP, 5 mL) was first equilibrated with five column volumes (CV) of binding buffer (PBS containing 10% (*v*/*v*) glycerol, 10× CMC detergent, and 20 mM imidazole, pH 7.6) at a flow rate of 1 mL/min. The supernatant containing AgrC–GFP was then loaded onto the column at a reduced flow rate of 0.5 mL/min to maximize the binding of the protein to the resin. The resins were then washed with the binding buffer to remove non-specifically bound proteins. The target protein was eluted using elution buffer (binding buffer containing 500 mM imidazole) through gradient elution. Purified AgrC–GFP was concentrated using an Amicon Ultrafree centrifugal filter (Millipore Corporation, Billerica, MA, USA) with a cutoff of 10 kDa, which simultaneously removed imidazole from the protein. The TEV digestion was performed at 16 °C for 5 h with a 10:1 TEV protease to fusion protein molar ratio. The digested sample was incubated with IMAC nickel resin and binding Buffer for 30 min. The flow-through containing the AgrC was subjected to size-exclusion chromatography at a flow rate of 1.0 mL/min on a Superdex-200 HiLoad 10/600 column (GE Healthcare, New York, NY, USA) that had been equilibrated with 10 mM HEPES buffer (pH 7.4) containing 100 mM NaCl, 10% glycerol and 10× CMC detergent. Protein concentration was determined using the BCA assay according to manufacturer’s instructions (Pierce, Rockland, IL, USA). The fractions containing the GFP-His6 tag in nickel resin were eluted.

### 3.8. Circular Dichroism (CD) Spectroscopy

CD was performed on a MOS-450 spectrometer (Bio-Logic, Alpes, France) with a 0.1 cm path length quartz cuvette. Samples were typically prepared in 10 mM HEPES pH 7.4 containing detergent at 10× CMC, 100 mM NaCl, and 10% glycerol. Spectra were obtained routinely at 20 °C. For measurements in the far UV range (190–250 nm) the concentration of AgrC was 0.5 mg/mL. Using sample volumes of 200 μL, 20 scans were acquired at standard sensitivity using an integration time of 1 s, a path length of 0.02 cm and a slit width of 0.5 mm that was equivalent to a 3 nm bandwidth. To determine the effects of AIP-I on the AgrC spectrum in this region, AIP-I (dissolved in 0.01% dimethylsulfoxide (DMSO)) was added at molar ratios of 0:1, 0.5:1, 1:1, and 5:1 AIP: AgrC with a 10-min incubation period following each addition of the samples before acquiring the spectra. DMSO exerted no significant effect on the AgrC spectrum, which was verified by subtraction of the spectrum that was obtained from a control sample that contained the maximum DMSO concentration. To investigate if AIP-I binding affects the thermal stability of AgrC, stabilized AgrC was incubated in the presence or absence of five-fold AIP-I at 20 °C, and spectra (208 nm and 220 nm) were obtained over a range of temperatures starting at 20 °C and increasing incrementally to 90 °C, with a 5-min equilibration period followed by a return to 20 °C at each step of the 20-min equilibration time.

## 4. Conclusions

Overexpression of membrane proteins remains a major challenge for biochemical and structural studies due to their partially hydrophobic surfaces and lack of stability. In this study, we assessed whether a GFP-containing fusion protein can be used to expedite the screening of protein expression systems. As our experimental model, we synthesized the membrane protein AgrC that was *C*-terminally fused to GFP. The GFP-moiety allowed for real-time monitoring of protein expression level using fluorescence detection. With whole-cell fluorescence measurements combined with in-gel fluorescence, western blot, and fluorescence microscopy methods, we screened multiple expression systems and identified the optimal combination to be that of C43 host cells, TEB culture medium, 20 °C assay temperature, and 0.1 mM IPTG for induction. More importantly, our results suggest that the use of the whole-cell fluorescence detection method provides a rapid evaluation of membrane protein expression levels with a limited investment in labor, which is in strong contrast to the traditional labor-intensive methods for overexpression screening using SDS-PAGE and western blot.

Detergents are an essential component for any membrane protein purification procedure and constitute a complicated variable that needs to be extensively investigated [23,32]. Detergent screening has important implications for protein stability, homogeneity, and activity. However, it is impossible to predict which detergent will be suitable for the extraction, purification, and retention of activity of a given membrane protein. We assessed the effect of different detergents on AgrC protein kinase activity and membrane solubilization. These experiments aimed to assess whether the overexpressed protein would be of suitable quality for further biophysical and biochemical analyses. We determined that the detergent Brij-35 was the optimal choice. In addition, we demonstrated that the level of AgrC autophosphorylation was significantly increased by five-fold in response to a two-fold molar excess of AIP-I. These results not only demonstrate a direct and specific interaction between the sensor kinase AgrC and its signaling molecule AIP-I but also confirm the potential of such an approach in future *in vitro* studies of signal recognition using other sensor kinases.

Far-UV CD analysis showed that AgrC in the presence of Brij-35 maintained its secondary structure. The CD spectra that we obtained were characteristic of a typical α-helical membrane protein (Figure 6A). Therefore, the purification method that we selected was suitable for obtaining correctly-folded protein with good structural integrity. Moreover, Figure 6B demonstrated that AIP-I exerted little effect on the secondary structural composition of AgrC. Figure 6C,D indicated that there was also no detectable effect of AIP-I on the thermal stability of AgrC. Further evaluation of the effect of different detergents on AgrC secondary structure is recommended.

The methods in this paper may be applied to the studies of other membrane proteins. Our data may serve as a strong basis for developing rational strategies to improve the quality and quantity of membrane protein preparations.

## Supplementary Information



## Figures and Tables

**Table 1:** Compositions of the culture media.

Number	Medium name	Medium component
1	LBE	0.5% (*w*/*v*) yeast extract, 1% (*w*/*v*) peptone, 0.5% (*w*/*v*) glucose, 1% (*w*/*v*) NaCl, 2 mM MgSO_4_, 20 mM KH_2_PO_4_, 80 mM K_2_HPO_4_
2	LBE-LP	0.5% (*w*/*v*) yeast extract, 1% (*w*/*v*) peptone, 0.1% (*w*/*v*) glucose, 0.5% (*w*/*v*) NaCl, 2 mM MgSO_4_, 5 mM KH_2_PO_4_, 20 mM K_2_HPO_4_
3	LBE-MP	0.5% (*w*/*v*) yeast extract, 1% (*w*/*v*) peptone, 0.5% (*w*/*v*) glucose, 0.07% (*w*/*v*) Na_2_SO_4_, 0.25% (*w*/*v*) NH_4_Cl, 2 mM MgSO_4_, 10 mM KH_2_PO_4_, 40 mM K_2_HPO_4_
4	2× TYE	1% (*w*/*v*) yeast extract, 1.6% (*w*/*v*) peptone, 0.5% (*w*/*v*) glucose, 0.5% (*w*/*v*) NaCl, 2 mM MgSO_4_, 20 mM KH_2_PO_4_, 80 mM K_2_HPO_4_
5	TBE	2.4 (*w*/*v*) yeast extract, 1.2% (*w*/*v*) peptone, 0.4% (*v*/*v*) glycerol, 2 mM MgSO_4_, 20 mM KH_2_PO_4_, 80 mM K_2_HPO_4_
6	32Y	3.2% (*w*/*v*) yeast extract, 0.8% (*w*/*v*) peptone and 0.58% (*w*/*v*) NaCl in 10 mM Tris–HCl pH 7.6
7	TB	2.4% (*w*/*v*) yeast extract, 1.2% (*w*/*v*) peptone, 0.4% (*v*/*v*) glycerol, 20 mM KH_2_PO_4_, 80 mM K_2_HPO_4_
8	4× TY	2% (*w*/*v*) yeast extract, 0.8% (*w*/*v*) peptone and 0.58% (*w*/*v*) NaCl

For the first five types of media, each culture contained a metal ion mixture (1 mM MnCl_2_•6H_2_O, 1 mM ZnSO_4_•7H_2_O, 0.2 mM CoCl_2_•6H_2_O, 0.2 mM NiCl_2_•6H_2_O, 0.1 mM FeCl_3_•6H_2_O).
