# Supplementary material for: Green Fluorescent Protein (GFP)-Based Overexpression Screening and Characterization of AgrC, a Receptor Protein of Quorum Sensing in Staphylococcus aureus
Source: Int J Mol Sci. 2013 Sep 6;14(9):18470–87. doi: 10.3390/ijms140918470 (PMC3794790; doi:10.3390/ijms140918470)
Supplement: Supplementary file 1 [file ijms-14-18470-s001.pdf]

Supplementary Information

Figure S1. Schematic representation of expression plasmid pET28a-AgrC-GFP.

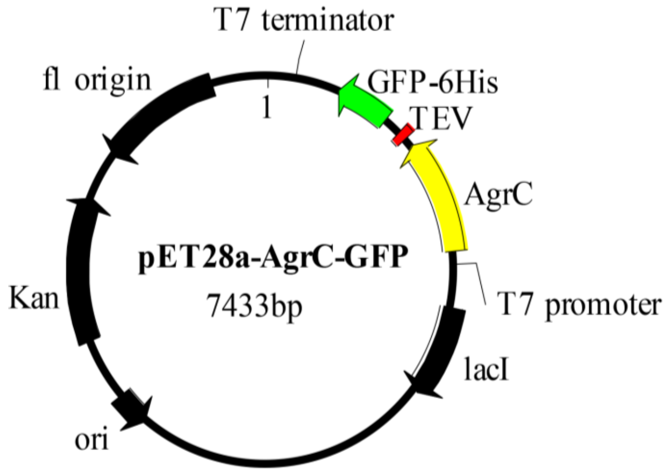

© 2013 by the authors; licensee MDPI, Basel, Switzerland. This article is an open access article distributed under the terms and conditions of the Creative Commons Attribution license (http://creativecommons.org/licenses/by/3.0/).
